# Co-Bioleaching of Pyrite Flotation Tailings and Crushed Printed Circuit Boards

**DOI:** 10.3390/molecules31060985

**Published:** 2026-03-15

**Authors:** Aleksandr Kolosoff, Vitaliy Melamud, Aleksandr Bulaev

**Affiliations:** Research Center of Biotechnology, Russian Academy of Sciences, 119071 Moscow, Russia; vmelamud.inmi@yandex.ru (V.M.); bulaev.inmi@yandex.ru (A.B.)

**Keywords:** biohydrometallurgy, acidophilic microorganisms, pyrite flotation tailings, printed circuit boards, e-waste recycling, community shifts

## Abstract

The aim of this study was to investigate the potential for co-bioleaching of ground printed circuit boards (PCBs) and flotation tailings using a single-stage biohydrometallurgical process. The ground PCB sample was a finely divided waste product from industrial shredding, which was collected using an air filtration system. The flotation tailings sample was mainly composed of pyrite (49%), quartz (29%), gypsum (8%), feldspar (8%), and chlorite (6%). The experiment was carried out in laboratory-scale reactors at 35 °C with constant aeration and a flotation tailings pulp density of 5% (solid-to-liquid ratio). In a control reactor, only flotation tailings were leached. In an experimental reactor, both flotation tailings and ground PCBs were leached simultaneously. The experiment was conducted in two stages. In the first stage, the experiment was carried out in a batch mode. The second stage involved two reactors operating continuously in cascade. During the experiment, we monitored the dynamics of several key parameters as a function of PCB concentration, including pH, redox potential, the concentrations of Fe^3+^ and Fe^2+^ ions, and the number of microbial cells. The 16S rRNA gene analysis revealed that the presence of PCBs had a significant effect on the composition of the microbial community. The concentration of PCB was gradually increased in order to examine the limits of the process and optimize potential economic benefits. The increase was done in 3 stages: 5 g/L in the first stage, from 5 to 12 g/L in the second stage, and up to 35.5 g/L in the third stage. However, this increase had a negative effect on the pyrite oxidation rate and the effectiveness of PCB bioleaching in continuous mode. The bioleaching efficiency of copper from printed circuit boards (PCBs) was above 70% in batch mode and above 80% in continuous mode at PCB concentrations up to 12 g per liter. Copper recovery decreased to around 53.1–61.6% as the PCB concentration continued to increase. The nickel leaching efficiency in batch mode was 46.3 ± 4.8%. In continuous mode, the nickel recovery decreased as the PCB concentration increased, reaching 48.53% in the first stage, then declining to 37.62% in the second stage and finally dropping to 27.06% in the third stage, depending on the higher concentration of PCB.

## 1. Introduction

Electronic waste (e-waste) has been identified as a promising source of non-ferrous and precious metals due to the high concentration of valuable components it contains [1,2]. This makes e-waste a significant resource for “urban mining”, potentially recovering metals and contributing to a circular economy [3,4]. However, the processing of this material is complicated by its chemical heterogeneity.

A specific challenge in biohydrometallurgical processes is the toxicity of certain elements in e-waste for acidophilic microorganisms, which are the main biological agents in these systems [5,6,7]. Unlike mineral raw materials, electronic waste is a complex and heterogeneous mixture that contains heavy metals, rare earth elements, and organic pollutants. These substances can inhibit microbial growth and metabolic activity, which in turn reduces the leaching efficiency [8,9,10].

Two main biotechnological approaches have been proposed as potential solutions to the challenge of metal toxicity in biorecovery. These approaches include two-stage bioleaching [11,12] and the co-processing of e-waste with sulfide minerals [13,14]. This study focuses the latter approach and investigates the one-step bioleaching process of a mixture pyritic flotation tailings and ground printed circuit boards.

The biooxidation of sulfide minerals, such as pyrite, arsenopyrite, chalcopyrite, and sphalerite, is a well-established technology in commercial biohydrometallurgy for extracting base and precious metals [15,16]. However, the sulfide ore flotation process generates tailings, which pose significant challenges for subsequent processing. For example, in Chile, one of the major copper producers, more than 500 million tons of tailings were produced in 2019 alone, and over 7 billion tons have been stockpiled over the past 30 years [17]. Tailings are known to present severe and long-term environmental risks, mainly due to their chemical instability and physical impact, and they require continuous management [18].

For an extraction process to be cost-effective, the value of the recovered metals must exceed the costs of processing. These costs include energy, reagents, equipment, and waste management. However, with current technologies, this balance is often negative for most tailings [19,20]. Bioleaching or acid leaching of low-grade tailings results in the production of pregnant leach solutions (PLSs) with very low concentrations of valuable metals. This poses a challenge when extracting these metals using conventional methods such as solvent extraction [19].

Heap leaching is limited by its dependence on climatic conditions. Microbial activity and chemical reaction rates are optimal within specific temperature ranges, which are difficult to maintain outdoors [21,22]. Furthermore, the variability in the granulometric composition of the feed material often results in poor and uneven percolation of the solution, leading to channeling of the solution and leaving valuable metals unextracted. These factors, combined with diffusion-controlled kinetics, lead to in extended processing times, which can take several years to achieve moderate metal recovery [22,23].

The joint bio-processing of electronic waste (e-waste) and flotation tailings is a novel approach that addresses two significant environmental concerns while enabling the sustainable recovery of metals. This integrated process improves the economic feasibility of processing low-grade waste materials, while also addressing the toxicity issues that can prevent efficient bioleaching of e-waste alone.

The fundamental mechanism behind this process mainly involves the chemical leaching of non-ferrous metals from printed circuit boards (PCBs) using products derived from the biooxidation of pyrite, which is found in mining waste. This process relies on acidophilic microorganisms that have the ability to oxidize sulfide minerals, particularly pyrite (FeS_2_), through well-established biochemical mechanisms that generate sulfuric acid and ferric ions as key metabolic products. Microorganisms oxidize ferrous iron (Fe^2+^) to ferric iron (Fe^3+^), and the resulting ferric ions then chemically attack sulfide minerals, regenerating ferrous ions that can be re-oxidized by microorganisms [24,25]. The simplified form that describes the chemistry behind the process of pyrite biooxidation can be represented by the following equations [25]:

2FeS_2_ + 7O_2_ + 2H_2_O → 2Fe^2+^+ 4SO_4_^2−^ + 4H^+^ (1)

4Fe^2+^ + O_2_ + 4H^+^ → 4Fe^3+^ + 2H_2_O (2)

FeS_2_ + 14Fe^3+^ + 8H_2_O → 15Fe^2+^+ 2SO_4_^2−^ + 16H^+^ (3)

The sulfuric acid and ferric ions generated through pyrite bio-oxidation act as potent lixiviants for extracting valuable non-ferrous metals from e-waste components, especially printed circuit boards (PCBs) [26,27]. This process involves several key reactions [26,27,28]:

Me^0^ + 2Fe^3+^ → Me^2+^ + 2Fe^2+^ (4)

2Me^0^ + 2H_2_SO_4_ + O_2_ → 2MeSO_4_ + 2H_2_O (5)

Although the use of pyrite in the recycling of PCB recycling has been discussed in the literature, most experiments have been conducted in laboratory settings using samples with high concentrations of copper [29,30,31]. Pyrite has been used in these studies as a substrate, but the specific mineral form of pyrite and the origins of the samples used have not been specified. The researchers have noted the lack of research on this topic and the limited experimental work, especially on PCB pulps with densities higher than 1%. It is essential to conduct further research on the combined bioleaching of crushed PCB and real-world objects containing pyrite, such as flotation tailings, in order to better understand the process.

The main objectives of this study were to investigate the feasibility and kinetics of pyrite biooxidation in the presence of printed circuit boards (PCBs), both in batch and continuous processes, using a laboratory-scale reactor. A major focus was placed on characterizing the specific changes in the microbial community caused by the addition of PCBs. Additionally, the study aimed to quantify the dynamics of copper and nickel leaching from PCBs and to determine the effect of PCB concentration on pyrite biooxidation and the recovery of non-ferrous metals.

## 2. Results

In accordance with the experimental design, the results are presented in two separate sections corresponding to the two operational modes we investigated. These sections describe the dynamics of key biooxidation parameters and include an analysis of associated shifts in microbial community structure.

### 2.1. Batch Mode Experiment Results

**Figure 1:**
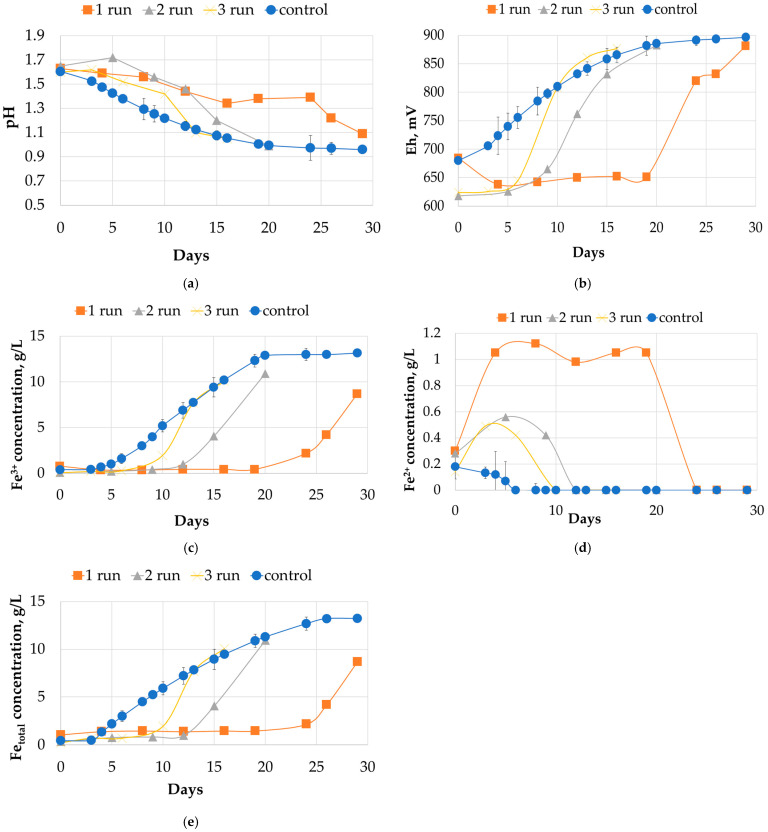
Changes in the liquid phase parameters during the experiments in a batch mode: (**a**)—pH; (**b**)—Eh (mV); (**c**)—concentration of Fe^3+^ ions (g/L); (**d**)—concentration of Fe^2+^ ions (g/L); (**e**)—total concentration of Fe^3+^ and Fe^2+^ ions (g/L). The error bars represent the standard deviation (SD) of the three replicates (*n* = 3) in the control reactor.

**Figure 2:**
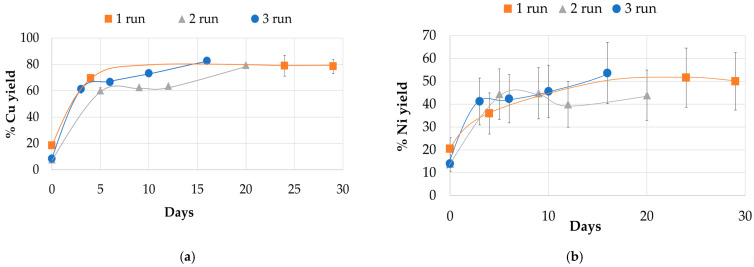
Yield of copper and nickel during the experiment in a batch mode: (**a**)—copper yield (% of Cu mass in PCB dust fraction) (**b**)—nickel yield (% of Ni mass in PCB dust fraction). The error bars represent the standard deviation (SD) of the measurements of metal concentrations in three repetitions (*n* = 3).

**Figure 3:**
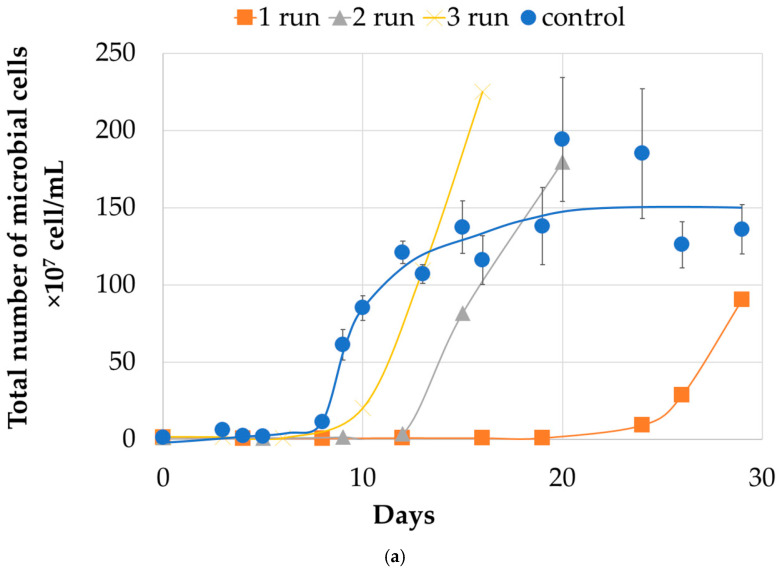

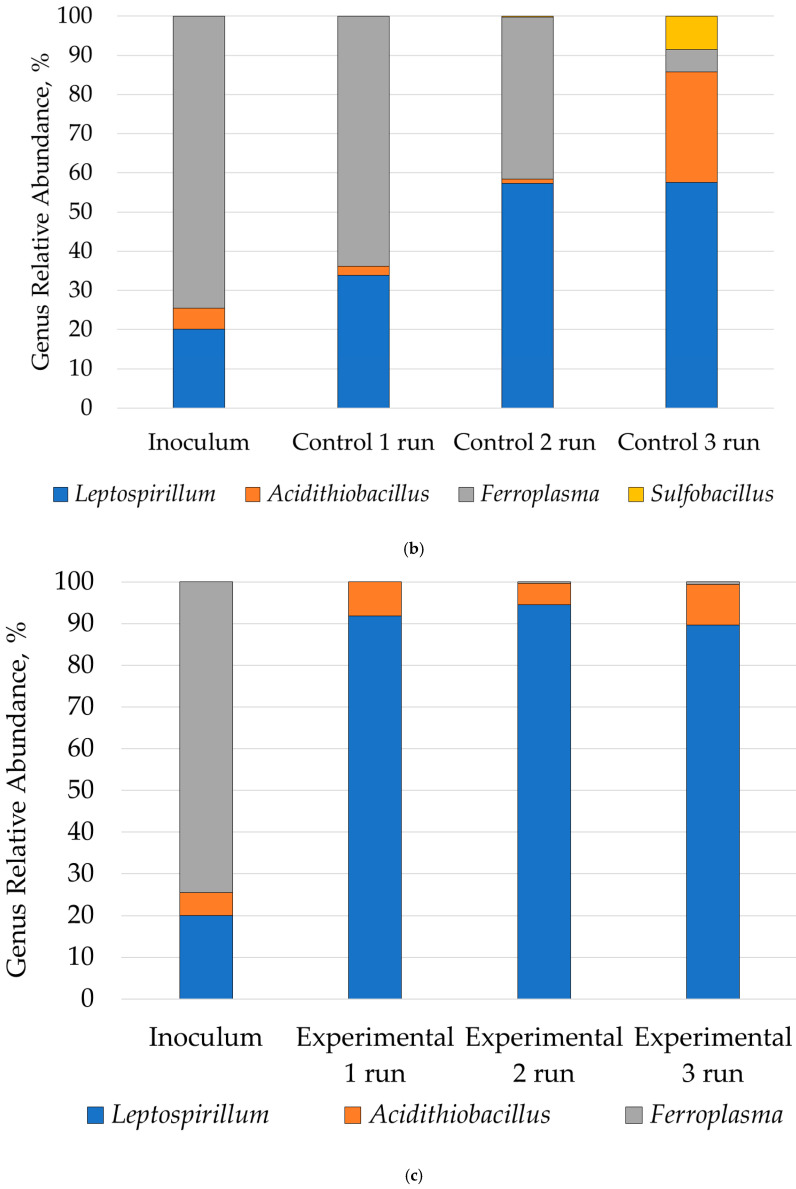
Analysis of microbial communities of the control and the experimental reactors during the experiment in a batch mode: (**a**)—total number of microbial cells (10^7^ cells/mL) in the experimental reactor during runs 1, 2, and 3, as well as the generalized dynamics in the control reactor over the same runs. The error bars represent the standard deviation (SD) of the three replicates (*n* = 3) in the control reactor.; results of molecular biological analysis of microbial populations: (**b**)—experimental reactor in the end of 1, 2 and 3 run; (**c**)—control reactor in the end of 1, 2 and 3 run.

During the experiment, we monitored changes in various parameters in a batch mode, including pH, redox potential (Eh), concentrations of ferrous and ferric iron, and total iron. We present the data from the experimental reactor during the first, second, and third runs in comparison to the average values from the control reactor over three runs. In the experimental reactor, we added 5 g/L of ground PCB to the initial pulp in all three runs. The results of the experiment are shown in Figure 1, Figure 2 and Figure 3, which illustrate the dynamics of the process parameters.

The results from the batch experiment are presented in Table 1. The table shows the time required for each run to reach the key process indicators: pH of 1.2, oxidation-reduction potential (Eh) of 800 mV, and complete oxidation of divalent iron (Fe^2+^) to undetectable levels in the liquid phase. With each subsequent run, the time to reach these values decreased. This trend was accompanied by a substantial increase in the number of microbial cells between runs, as indicated by the acceleration of the process. The selected reference values for pH, Eh, and Fe^2+^ were based on stabilized parameters observed in control reactor and are consistent with the thresholds established in our previous research [32,33,34].

#### 2.1.1. Liquid Phase Analysis

Figure 1 shows the changes in liquid-phase parameters during an experiment conducted in a batch mode. The curves demonstrate that the dynamics of these changes varied from the first run to the third run, approaching the results obtained in the control reactor. Analysis of pH values (Figure 1a) revealed distinct trends between the experimental and control reactors. During the first 24-day run in the experimental reactor, the pH remained relatively stable, after which it decreased rapidly. This period of stability was significantly shorter during the second experiment, lasting approximately 12 days before a similar decrease was observed. In contrast, during the third experiment, the pH in the experimental reactor started to decrease immediately from the initial point of cultivation, converging with the levels recorded in the control reactor within a 12-day period. The data obtained from the control reactor over the three runs did not reveal a comparable extended period of pH stability. Instead, the pH values showed a consistent decreasing trend throughout each cultivation cycle. It is worth noting that the addition of sulfuric acid was not intended to clearly track the moment of acidification due to pyrite oxidation.

Changes in the oxidation-reduction potential (Eh) (Figure 1b), which serves as an indicator of iron ion oxidation activity in the medium, showed a clear correlation with the pH trends. During the first experimental run, the Eh value increased rapidly between days 19 and 24 of cultivation, reaching 820 mV on day 24. After that, the potential continued to increase thereafter, attaining a final value of 881 mV after the complete 29-day biooxidation period was completed. During the second run, the increase in Eh began earlier, after 9 days, reaching 832 mV on day 15 and continuing to a final value of 883 mV at the end of the 20-day period. In the third run, Eh values remained at the initial baseline for six days, after which an increase to 806 mV was observed. This value exceeded the average Eh measured in the control reactor at the corresponding time point. By the end of the third run, the Eh in the experimental reactor had reached 877 mV. The dynamics of Eh in the control reactor showed a more gradual and linear increase during each run, without the sharp, stepwise changes observed in the experimental system. There was no significant stabilization period at the initial Eh level in the control, and the final Eh values stabilized within a consistent range of 870 to 890 mV at the end of each run in the control reactor.

The diagrams in Figure 1c,d, and e show the dynamics of iron ion concentrations. In the first experiment, there was no significant increase in the concentration of trivalent iron (Fe^3+^) until days 24–26 of the cultivation process. During this initial phase, the concentration of divalent iron (Fe^2+^) remained relatively high and stable. It is worth noting that during the first experiment, the maximum possible concentration of total iron in the liquid phase was not reached within the 29-day period in the experimental reactor. During the second run, Fe^2+^ concentrations were still detectable in the liquid phase up until day 12. The concentrations of Fe^3+^ and total iron remained relatively stable until that point. After day 12, there was a decrease in the concentration of Fe^2+^ below the detection limit, which coincided a sharp increase in the concentration of Fe^3+^ ions. By the third run, Fe^2+^ had become undetectable by day 10. A significant increase in Fe^3+^ and total iron concentrations followed, producing dynamics of iron oxidation and mobilization similar to those in the control reactor. However, the dynamics of iron solubilization from the pyritic flotation tailings in the control reactor and the rate of Eh change, were distinct from those in experimental runs. Composite data from all three control runs showed that the concentration of Fe^2+^ was consistently lower than in any experimental run and was not detected in the liquid phase after the first six days of biooxidation in any control run. Increases in Fe^3+^ and total iron concentrations in the control reactor followed a nearly linear trajectory, eventually exceeding the level of 10 g/L in all runs.

Figure 2 summarizes the mobilization of copper (Figure 2a) and nickel (Figure 2b) into the liquid phase during runs 1–3 in the experimental reactor. A significant fraction of copper was solubilized within the initial hours of processing. Analysis of samples taken 30 min after incubation showed that 10–20% of the total copper content from the printed circuit board sample had been extracted. Copper recovery exceeded 70% of the initial mass of copper in the PCB dust fraction by the end of leaching in all runs, with no major differences observed in the overall extraction dynamics. Nickel extraction followed a similar pattern, characterized by a rapid increase in concentration within the first 30 min. Subsequently, nickel mobilization proceeded with comparable kinetics across all three runs, with final recovery rates ranging from of 50.0% (1st run), 44.0% (2nd run) and 53.6% (3rd run).

#### 2.1.2. Microbial Population Analysis

Analysis of the microbial population demonstrated a strong correlation between the liquid phase parameters during the experiment (Figure 3a). It is also revealed a progressive shift in the experimental reactor from the first to third run. The lag phase for microbial cell growth was 24 days in the first run of the experiment, correlating with the initiation of iron oxidation and a decrease in pH. This phase was significantly shorter in the second run (12 days), after which rapid growth occurred, reaching control values by day 20. In the third run, the lag phase continued to decrease (10 days) with growth reaching control levels by day 12. The dynamics of microbial growth followed a classic model for batch culture in the control reactor, with a lag phase characterized by a low steady growth rate for 6–8 days, followed by a logarithmic phase between days 8–11, and finally a plateau with a cell concentration of 130–160 × 10^7^ cells/mL.

Analysis of the inoculum revealed that the relative abundance of the microorganisms was dominated by the acidophilic archaea of the genus *Ferroplasma*, mesophilic bacteria of the genera *Leptospirillum* and *Acidithiobacillus*, and a minor group of bacteria of the genus *Sulfobacillus*.

The addition of the PCB dust fraction resulted in the formation of distinct communities from the same inoculum. In the experimental reactor (Figure 3b), the community that developed during the first run remained stable throughout the second and third runs. Mesophilic bacteria of the genera *Leptospirillum* and *Acidithiobacillus* were the predominant microorganisms at the end of all three runs. Consortia from runs 2 and 3 also included archaea of the genus *Ferroplasma*, although at relatively low abundances (0.35% and 0.62%, respectively).

The population shift in the control reactor (Figure 3c) showed an increase in the abundance of *Leptospirillum* and *Acidithiobacillus*, accompanied by a decrease in *Ferroplasma* from the first to the third run. Additionally, there was an increase in bacteria belonging to the genus *Sulfobacillus*.

### 2.2. Continuous Mode Experiment Results

**Figure 4:**
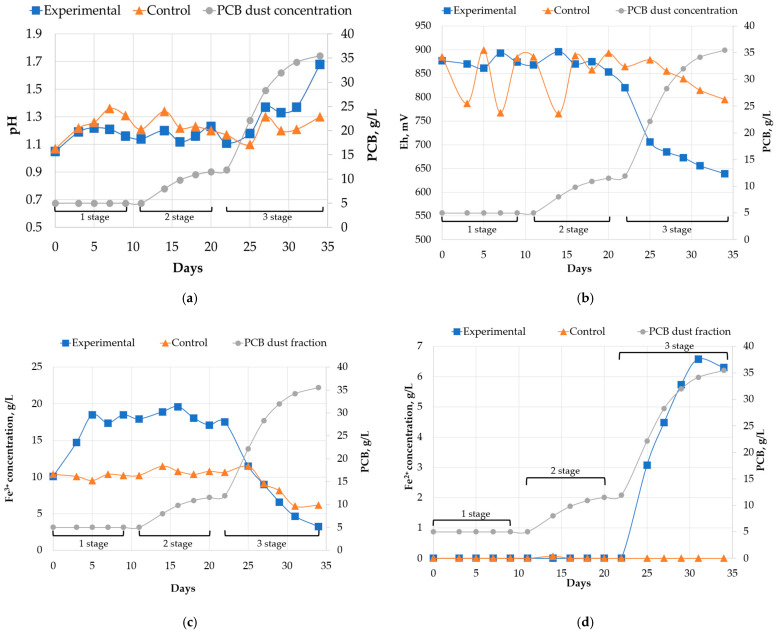

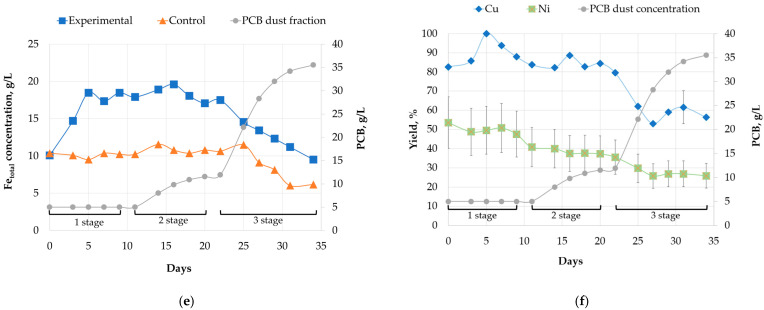
Changes in the liquid phase parameters in response to increasing PCB dust concentration during experiment in continuous mode: (**a**)—pH; (**b**)—Eh; (**c**)—concentration of Fe^3+^ ions (g/L); (**d**)—concentration of Fe^2+^ ions (g/L); (**e**)—total concentration of Fe^3+^ and Fe^2+^ ions (g/L), (**f**)—yield of Cu and Ni. The error bars represent the standard deviation (SD) of the measurements of metal concentrations in three repetitions (*n* = 3).

**Figure 5:**
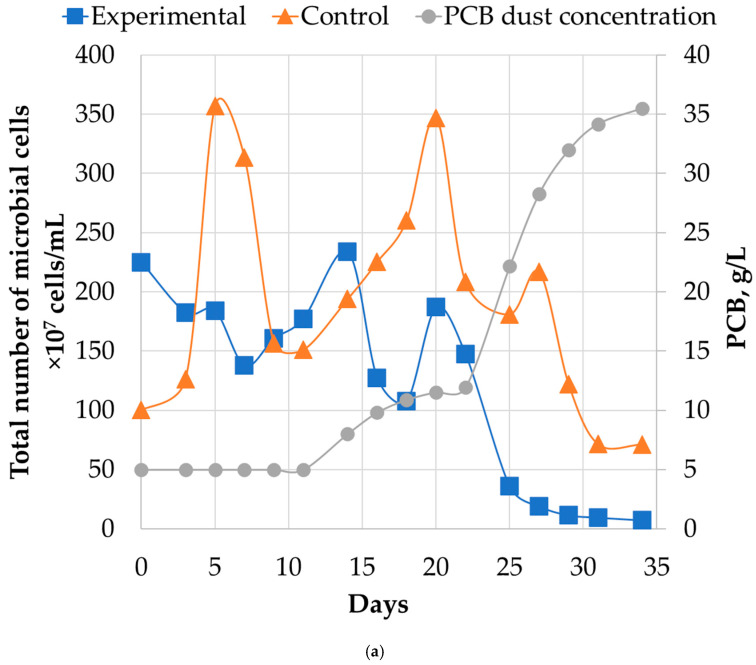

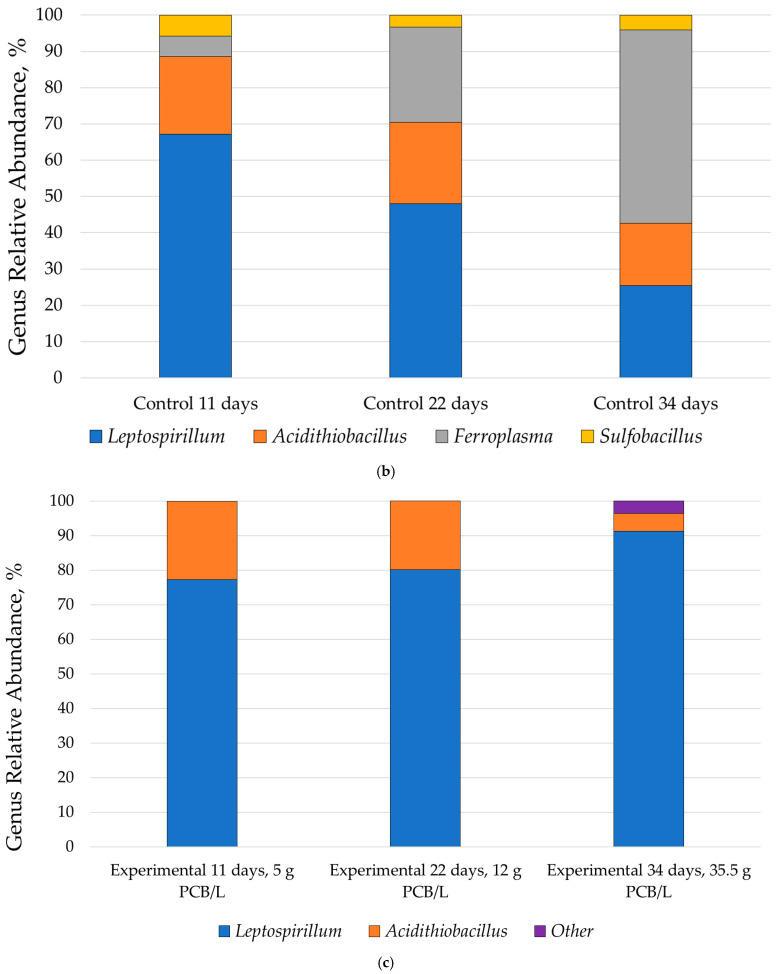

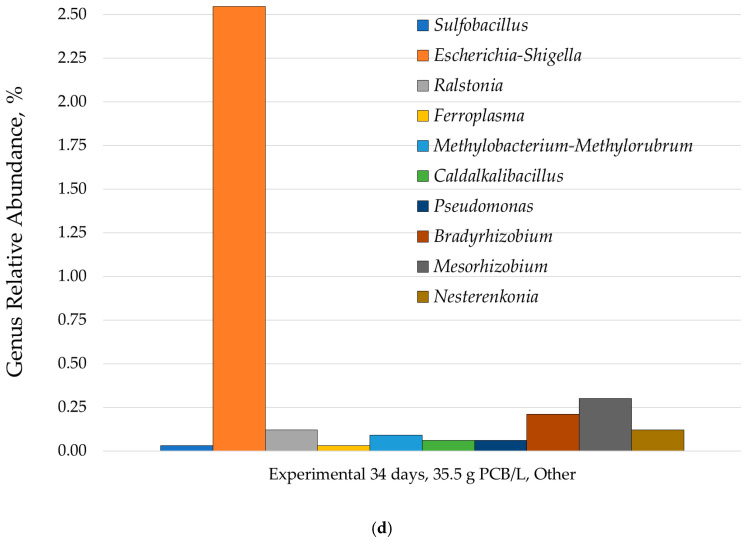
Dynamics of microbial abundance and community structure under continuous leaching conditions: (**a**)—total microbial cell counts. (**b**)—population composition in the control reactor. (**c**) —community shifts in the PCB-fed experimental reactor. (**d**)—detailed composition of the “Other” taxa in the community analysis of the experimental reactor on 34th day.

After the batch leaching phase of the third run, the experiment continued by switching the reactors to continuous mode. The concentration of PCB dust fraction in the leaching medium was gradually increased. This study aimed to investigate the effects of PCB pulp density on both bioleaching efficiency and the composition of the associated microbial community. The resulting dynamics of key leaching parameters are illustrated in Figure 4 and Figure 5.

The results of the continuous mode experiments are presented in Table 2. This table includes average liquid-phase parameters, such as pH, oxidation-reduction potential, Fe^3+^ and Fe^2+^ concentrations, microbial cell counts, and extraction yields for copper and nickel at each stage of the experiment. This structured presentation allows us to track the evolution of solution chemistry and biological activity as the printed circuit board density increases. The data corresponds directly to the three operational phases of the continuous reactor run. The confidence interval was calculated using data obtained from both the control and experimental reactors during the three stages of the experiment, which each lasted 11–12 days.

#### 2.2.1. Liquid Phase Analysis

The pH values in both the control and experimental reactors remained at comparable levels during stages 1 and 2 (Figure 4a). Following the initiation of continuous flow, the pH gradually increased to values of 1.3 and 1.1 in the control and experimental reactors, respectively. On the 25th day of the experiment (stage 3), the pH in the control reactor decreased below that of the experimental reactor. This trend that persisted until the end of the experiment, while the pH in the experimental reactor increased sharply in response to the rising concentration of PCB in the pulp during the third stage. As a result, the final pH value in the experimental reactor was 1.68, whereas no sulfuric acid was added to adjust the pH during the experiment.

Both Eh curves in Figure 4b remained relatively stable through day 20, which corresponds to the end of the second experimental phase. After that, the Eh level in the control reactor varied between 750 and 900 mV, while the Eh in the experimental reactor consistently stayed below 750 mV. As pulp density increased during the transition to the third phase, the Eh profiles of the two reactors began to diverge. During this final phase, both reactors experienced a gradual decrease in Eh, with the control reactor reaching 795 mV and the experimental reactor reaching 639 mV by the end of the experiment.

During operation in continuous mode, shifts in ferric (Fe^3+^) and ferrous (Fe^2+^) iron concentrations followed patterns consistent with those observed for pH and Eh. Throughout the first and second stages, the concentration of Fe^2+^ in the liquid phase of both reactors was below the detection limit of the titrimetric method, with total iron comprising Fe^3+^ ions. The total iron concentration was higher in the experimental reactor due to the addition of iron-containing PCB material and pyrite flotation tailings. During the third stage, the Fe^3+^ concentration in the experimental reactor first decreased to match that of the control reactor, then decreased further to 3.22 g/L. Concurrently, the Fe^3+^ concentration in the control reactor decreased to 6.0–6.5 g/L, while its Fe^2+^ concentration was undetectable. In contrast, the Fe^2+^ concentration in the experimental reactor significantly increased during this stage, correlated with the observed decrease in redox potential. This demonstrates that increasing the printed circuit board pulp density above 1.2 g/L is linked to a decrease in both Eh and Fe^3+^ concentration, resulting in the accumulation of Fe^2+^ in the solution.

The increase in PCB pulp density had a negative impact on the leaching rates of copper and nickel (Figure 4f). Copper extraction remained relatively high until day 22 of the experiment, exceeding 80%. This was consistent with the concurrent trends in Eh, pH, and ferric iron concentration, which also remained stable. A further increase in PCB concentration led to a decline in copper extraction. A plateau in this decline, corresponding to a 55–60% mass yield of copper, was observed at a PCB concentration range of 28–35.5 g/L. Nickel yield also decreased with increasing PCB density, following a similar pattern to that of copper. The pattern of nickel extraction decline corresponded with each of the three experimental stages. The average nickel extraction was 48.53% during the first stage, declining to 37.62% in the second stage, and finally dropping to 27.06% in the third stage.

#### 2.2.2. Microbial Population Analysis

The dynamics of the total microbial cell count in the experimental reactor followed a trend similar to the key biooxidation process parameters. During the first and second stages, the total cell count was comparable to that in the control reactor, oscillating within the range of 150–250 × 10^7^ cells/L in the first stage and 100–200 × 10^7^ cells/L in the second stage. However, a sharp decrease in cell count was observed in the third stage, corresponding to an increase in PCB dust concentration. The final cell count reached a value of 7.37 × 10^7^ cells/mL after this stage.

The community shift tendencies observed during the batch stage persisted throughout the continuous mode. The predominance of the mesophilic bacteria *Leptospirillum* and *Acidithiobacillus* remained during stages 1 and 2, although there was a slight shift in stage 3. Along with the sharp decrease in cell count, the microbial community analysis revealed the presence of a diverse group of minor soil bacteria, including the genera *Escherichia-Shigella* (2.55% relative abundance), *Ralstonia* (0.12%), *Methylobacterium-Methylorubrum* (0.09%), *Caldalkalibacillus* (0.06%), *Pseudomonas* (0.06%), *Bradyrhizobium* (0.21%), *Mesorhizobium* (0.3%), and *Nesterenkonia* (0.12%). Genera *Sulfobacillus* and *Ferroplasma*, which were abundant in the liquid phase of the control reactor, were not detected in the experimental reactor during the first and second stages. However, they were detected at the end of stage 3, with relative abundances of 0.03%.

Metabarcoding analysis of the microbial community in the control reactor during continuous mode revealed dynamic population shifts. Contrary to the declining trend observed during batch operation, the relative abundance of the genus *Ferroplasma* increased throughout the continuous process, rising from 5.6% on day 11 to 26.2% on day 22 and reaching 53.2% by the end of the experiment. This increase was accompanied by a proportional decrease in the abundance of the genus *Leptospirillum*. Bacteria of the genera *Sulfobacillus* and *Acidithiobacillus* were present throughout the continuous experiment. The relative abundance of *Sulfobacillus* remained in the range of 3.3–5.8%, while the abundance of *Acidithiobacillus* ranged from 17.2 to 22.4%.

Direct cell counting in the liquid phase of the control reactor showed oscillations in microbial number with a relatively high amplitude, ranging from 100–350 × 10^7^ cells/mL, without a clear pattern. After 25 days, the microbial concentration declined to values of 71–72 × 10^7^ cells/mL, coinciding with a decrease in ferric iron concentration in the liquid.

The analysis presented data on the two connected communities in the reactor cascade. It showed that both communities had distinct structures and patterns of change, even though there was a constant flow of microorganisms between the control reactor and the experimental reactor.

## 3. Discussion

The results from both stages of the experiment indicate that the oxidation of pyrite and ferrous iron in the presence of PCB dust may be challenging. In particular, the attempt to carry out joint pyrite and PCB bioleaching without prior microbial adaptation was hindered by a significant delay phase and relatively low oxidative activity. However, chemical leaching of copper and nickel in batch mode was relatively high and did not seem to depend on bioleaching parameters. These findings will be discussed separately.

### 3.1. Experiment in a Batch Mode

The results from the batch experiments demonstrate a clear trend of microbial adaptation to the toxic environment of printed circuit board materials. This is shown by the gradual convergence of key parameters (pH, ferrous iron concentration, and cell count) in the experimental reactor towards control values from first to third run.

The initial cycle was characterized by a significant deviation in all parameters and an extended lag phase exceeding 20 days. This can be attributed to the stress response to the toxic components of the PCB sample. During the first cycle, a gradual decrease in pH was observed in the experimental reactor, from 1.63 to 1.38 over the first 20 days (Figure 1a). This suggests that the process of oxidation of sulfide sulfur was occurring, without a corresponding increase in the number of microbial cells or oxidation of ferrous iron being observed.

This trend suggests that the mineral matrix of pyrite, present in the flotation tailings, was central to the adaptation process, functioning as both an energy substrate and a protective habitat [35,36].

Pyrite may serve a dual purpose. Firstly, its continuous oxidation provides a steady supply of ferrous iron, acting as an energy source for iron-oxidizing microorganisms even under inhibitory conditions. Secondly, the surface of pyrite acts as a platform for the formation of protective biofilms. These structured environments mitigate toxicity by reducing direct contact with complex organic substances released from PCB dissolution, promoting not only the survival, but also the gradual restructuring of the microbial community. Chemically, PCB dust served as an additional source of iron. This is evident by the distinctive pattern of biooxidation parameter dynamics in its presence. Specifically, there was a constant presence of ferrous iron in solution during the initial lag phase at concentrations of 0.7 g/L in the first run, 0.28 g/L in the second run, and 0.14 g/L in the third run (Figure 1c,d). This pattern can occur as a result of the rapid acidic leaching of iron contained within the PCB sample, as well as the leaching of non-ferrous metals with ferric ions. This assumption can be confirmed by the dependence between the initial concentration of ferric iron provided with the inoculum. In contrast, the more linear trend of pyrite oxidation observed in the control reactor is consistent with the established mechanism of chemical and biological sulfide mineral leaching. This difference in the initial iron release kinetics has created a distinct chemical baseline that has further complicated the microbial response in the experimental system.

A significant consequence of PCB toxicity was a major change in community composition, which explains the prolonged lag phase and slower initial oxidation rates. Several studies have reported the negative impact on acidophilic microbial communities [5,6,37]. The experiment with solid-phase PCBs with pulp densities of 1 and 2% showed a rather negative effect on acidophilic microorganisms, with *L. ferriphilum* and *S. benefaciens* as dominant species [37]. The unadapted culture did not show significant growth in the presence of PCB, neither at 1% nor 2% pulp density. The adapted culture was able to grow in the presence of PCB, but the short observation period did not allow to clearly determine its dynamics. Researchers confirm that PCBs contain high concentrations of toxic heavy metals, such as copper, which can significantly inhibit iron-oxidizing processes at levels found in PCB leachates. Additionally, PCBs contain toxic organic compounds, including polybrominated biphenyls (BFRs) used as flame retardants, which have also been shown to inhibit bacterial growth [38]. However, the specific toxicity of these BFRs towards certain bacteria requires further investigation. The extreme acidity of the medium may have accelerated the dissolution of these toxic metals, potentially leading to the leaching of brominated flame retardants (BFRs) from the polychlorinated biphenyls (PCBs), creating an extremely hostile environment.

Individually, high concentrations of metals do not significantly affect the growth of acidophilic microorganisms, such as *Acidithiobacillus*, *Ferroplasma*, and *Leptosirillum*. This has been shown in several studies [32,33,37]. In particular, a leaching solution containing at least 15 g of copper per liter was used in the study [37]. When diluted in five volumes of medium (3 g/L copper and other metal concentrations), this solution did not decrease the oxidation rate of ferrous iron. However, these concentrations of non-ferrous metals were not achieved during the experiment, and the main conclusion of the study was about the toxicity of plastic PCB parts, which were present in significant amounts in the experimental medium.

Efficient pyrite biooxidation often relies on synergistic interactions between *Ferroplasma*, which excels at initial attachment, and *Leptospirillum*, a highly efficient iron oxidizer [39]. The increased lag phase observed in the process is likely due to the inhibitory effect of PCB leachates on *Ferroplasma*. This disruption of the synergy has left a community dominated by *Leptospirillum* that requires time for functional adaptation. This scenario has been exacerbated by the loss of organic carbon detoxification, as low-molecular-weight organic acids can inhibit chemolithotrophic organisms by disrupting their pH homeostasis [40]. These acids are typically metabolized by mixotrophic organisms such as *Ferroplasma* and *Sulfobacillus*, which help detoxify the microenvironment [34,41]. The acute suppression of *Ferroplasma* has deprived the community of this important function. Consequently, *Leptospirillum* faced both direct PCB toxicity and an accumulating burden of inhibitory metabolites. The subsequent detection of a resilient *Ferroplasma* sub-population (0.35% in run 2; 0.62% in run 3) in protected microniches correlates with improved leaching dynamics. This suggests that the partial restoration of detoxifying synergy was key to adaptation.

This interpretation is supported by comparing the different fates of *Ferroplasma* in the two reactor systems. Although both systems showed a decrease in its relative abundance, the underlying reasons for this were fundamentally different. In the experimental reactor, *Ferroplasma* could be rapidly suppressed to near-negligible levels by the PCB dust toxicity, an effect that probably also affected other mixotrophs and heterotrophs. In contrast, its gradual decline in the control reactor represented a natural succession, where its niche was competitively occupied by *Sulfobacillus* due to resource competition. This critical comparison confirms that PCB materials directly inhibited *Ferroplasma*, specifically disrupting the *Ferroplasma*-*Leptospirillum* interaction and crippling initial biooxidation kinetics.

In summary, the batch experiments reveal that pyrite acted as a critical ecological niche. A key challenge in co-processing e-waste is the disruption of essential microbial synergies. System recovery depended on the slow re-establishment of a detoxifying, mixotrophic functionality within a tolerant, bacterial-dominated framework, underscoring the paramount importance of community interaction for process efficiency.

### 3.2. Experiment in Continuous Mode

During the first and second stages of the continuous experiment (days 0–22), the key leaching parameters—specifically, ferrous iron concentration, pH, and redox potential (Eh)—in the experimental reactor with printed circuit board (PCB) material remained comparable to those in the control reactor, even as the PCB pulp density was increased from 0.5% to 1.2%. In contrast, the microbial cell count in the PCB reactor showed a different pattern. From approximately days 10 to 14, the cell number was higher than in the control. However, after a further increase in PCB pulp density, the cell count began to decline and subsequently remained stable at a level lower than that of the control reactor until the end of the experiment.

The cell concentration in this two-stage cascade system can be described by specific relationships. In the first (control) reactor, which received only pyritic flotation tailings, the cell concentration depends on the growth rate of the microbial community. In the second (experimental) reactor, which received tailings and PCB dust, the cell concentration is influenced by two factors: the growth rate within this reactor and the continuous inflow of cells from the control reactor. This relationship can be expressed by the following system of Equation (6):

(6) $$\left\{\begin{array}{c} \frac{dX_{1}}{d\tau }=X_{1}\left(\mu_{1}-D\right)\\ \frac{dX_{2}}{d\tau }=DX_{1}+\mu_{2}X_{2}-DX_{2\;} \end{array}\right\}$$

Here, $\mu_{1}$ and $\mu_{2}$ are the specific growth rates in reactor 1 and reactor 2, $X_{1}$ and $X_{2}$ are the cell concentrations, and D is the dilution rate (which are the same for both reactors). Since we are interested in examining the cell count in the experimental reactor relative to the control re-actor, we assume the system to be at steady state for reactor 1, where $\frac{dX_{1}}{d\tau }$ = 0 and therefore $\mu_{1}$ = D and $X_{1}$ is constant. This is a fundamental property of chemostat operation [42]. The solution for the system reduces to the following function for the cell concentration in reactor 2:

(7) $$X_{2}\left(\tau \right)=\;\frac{DX_{1}}{D-\mu_{2}}+Ce^{\left(\mu_{2}-D\right)\tau },$$

where *C* is a constant from integration. The long-term behavior of $X_{2}$, assuming a constant $X_{1}$, depends on the value of the growth rate $\mu_{2}$:

If $\mu_{2}$ > D > 0, the cell concentration $X_{2}$ will grow exponentially.

If D > $\mu_{2}\;$> 0, *X_2_* will stabilize at a level higher than $X_{1}$.

If $\mu_{2}$ = 0, $X_{2}$ will eventually equal $X_{1}$.

Only if $\mu_{2}\;$ < 0 can the steady-state concentration $X_{2}$ become lower than $X_{1}$.

Our experimental data show an initial phase (days~10–14) where $X_{2}$ remained within the close range to $X_{1}$, consistent with a temporary period of growth rate close to zero ($\mu_{2}$~0). Following the increase in PCB pulp density, $X_{2}$ declined and stabilized at a level below $X_{1}$. According to the model, this final, sustained deficit can only be explained by a negative specific growth rate ($\mu_{2}$ < 0) in the experimental reactor.

This indicates that the higher PCB loading caused significant toxicity, causing cell death to exceed growth, despite the continuous supply of cells from the control reactor. Previous studies have documented the toxic effect of PCB components on acidophilic bioleaching microorganisms [43].

**Figure 6:**
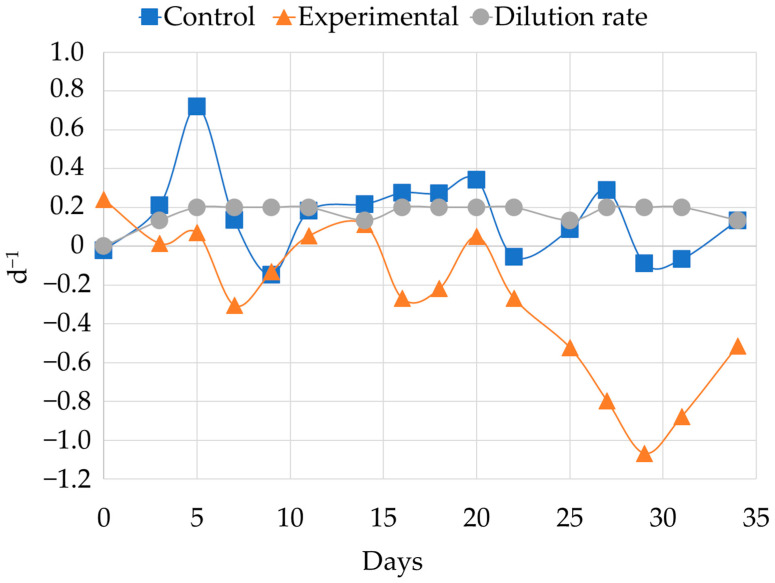
Growth rate in control and experimental reactor during the experiment in continuous mode.

In order to validate the model qualitatively, we calculated the growth rate of microbial cells in both the control and experimental reactors. The dynamics of the changes in growth rate during the three stages of the experiment are shown in the diagram below (Figure 6).

The data presented in the diagram indicates that the growth rate of the experimental reactor during the first 20 days of the experiment (stages 1 and 2) fluctuated around zero. As the concentration of PCB increased, the growth rate significantly decreased, reaching −1.07 days^−1^, which may indicate cell death. In contrast, the growth rate in the control reactor remained stable at a level determined by the dilution rate, confirming the assumption that the chemostat model can be used.

The parameters of biooxidation observed in the experiment were strongly influenced by the cell count in the experimental reactor. Despite the fact that no specific growth of microbial cell quantity was observed in the experimental reactor, the oxidation of ferrous iron was maintained stable during the first and second stages of the experiment. The microbial population analysis also revealed that the growth rate in the second reactor was affected by the relative abundance of different microbial genera in the control reactor. By the end of the first stage of the experiment (day 11), the microbial community in the control reactor was dominated by two mesophilic bacterial genera: *Leptospirillum* and *Acidithiobacillus* (67.2% and 21.4% of relative abundance, respectively). The same genera were dominant in the experimental reactor, whereas *Ferroplasma* and *Sulfobacillus*, which were fed to the experimental reactor continuously with the control reactor’s liquid phase, were not found in the community of the experimental reactor. The trend became even more clear from the results of the microbial population analysis at the end of stages 2 and 3. While the archaea of the genus *Ferroplasma* reached a more dominant role in the control reactor community (26.2% at the end of the second stage and 53.2% at the end of the third), the gap between the cell count in the control reactor and that in the experimental reactor progressed. It was also shown that *Sulfobacillus* and *Ferroplasma* were not able to become a significant part of the microbial community of the experimental reactor during the second and third stages. This observation can only be explained by the permanent cell death of *Ferroplasma* and *Sulfobacillus* in the presence of the PCB dust sample in the leaching medium. This is also correlated with mathematical modeling. These two genera are known to be important iron-oxidizers in bioleaching systems [44,45]. This cell death can lead not only to the decline of cells capable of iron or pyrite oxidation but also to the accumulation of lysis products. It has been reported that these lysis products, such as organic acids, can cause the inhibition of autotrophic bacteria and potentially further cell death [40,41]. These significant factors, or a combination of them, lead to a decrease in overall process efficiency.

The analysis of the microbial community in the experimental reactor at the end of the third stage revealed the presence of different soil bacteria. The genera *Ralstonia* (0.12%), *Methylobacterium Methylorubrum* (0.09%), *Caldalkalibacillus* (0.06%), *Pseudomonas* (0.06%), *Bradyrhizobium* (0.21%), *Mesorhizobium* (0.3%), and *Nesterenkonia* (0.12%) are known to be predominantly mesophilic neutrophilic or moderately alkaliphilic bacteria and are not able to maintain their quantity in the harsh conditions of a bioleaching reactor of low (1.0–2.0) pH. This phenomenon probably occurred because of soil particles in the PCB dust contained, as it was not sterilized before the experiment, and was accompanied by the potent lysis of the microbial cells in the experimental reactor. However, the presence of these bacteria in the bioleaching process is not expected to play a significant role in acidophilic metabolic chains, as they have low adaptability to low pH conditions.

During the experiment in continuous mode, the extraction of copper and nickel was found to be dependent on the PCB dust concentration in the pulp. Copper leaching exceeding 80% during the first and second stages of the experiment was well supported by the microbial production of ferric iron. Since the ferric concentration in the experimental reactor was higher than that in the control reactor until day 25 of the experiment, pyrite and PCB-originated iron oxidation in the presence of PCB took place. A decrease in copper yield can be caused by the decline of the oxidation of ferrous iron by microorganisms, as the kinetics of chemical ferric iron-driven leaching slowed.

In contrast, the extraction of nickel decreased proportionally as the PCB dust concentration increased. Nickel in printed circuit boards is primarily found in the form of a thin protective layer, which is made of metallic nickel or a nickel-phosphorus This layer acts as a barrier to ensure corrosion resistance, solderability, and mechanical strength of the contact pads. Nickel can be found on contact pads, lamellas, and metallized holes in printed circuit boards. These areas are part of the intermediate coating layers that provide conductivity and protection. Additionally, nickel is used in microcircuit terminals, transistors, and connectors to provide a nickel sublayer coating before finishing, as well as to enhance the wear resistance of connector plugs. Nickel is mostly presented in its metallic form in PCB and the leaching process of this metal can be described according to the chemical equation [26] (8):

Ni^0^ + ½ O_2_ + H_2_SO_4_ → NiSO_4_ + H_2_O (8)

Acidic leaching occurs under reducing conditions at −0.25 V Eh. This means that the leaching of nickel is not necessarily related to the ferric concentration, but rather controlled by the diffusion of H^+^, which can also be described by the shrinking core model [26,46]. Therefore, it was hypothesized that nickel oxidation would not directly correlate with the biooxidation process, but would only be influenced by the residence time in the sulfuric acid environment. However, the experiment revealed a decrease in leaching efficiency. This could be attributed to the fact that at higher PCB concentrations, the acid produced during pyrite oxidation interacted with Fe^0^ present in the boards.

In summary, it is important to note that pyrite oxidation during the joint leaching process with printed circuit boards is significant due to the potential application of this method as part of a sulfide oxidation process, which is widely used in industrial applications.

## 4. Materials and Methods

### 4.1. Old Flotation Tailings Sample and Its Pretreatment

During the experiments, we used a sample of old pyrite flotation tailings obtained from a copper-zinc ore from an industrial processing plant (Uchalinsky GOK, Uchaly, Republic of Bashkortostan, Russia). This sample had previously been used in our research on the innovative complex process of chemical leaching [47]. The sample contained pyrite (49%), quartz (29%), gypsum (8%), feldspar (8%), chlorite (6%) and a relatively small amount of sphalerite and chalcopyrite (both less than 0.5%). The total iron content was 29.5%, the total sulfur content was 29.2% and the sulfide sulfur content was 25.4%. The copper content was 0.12% and zinc content was 0.26%.

It has previously been shown that the distilled water pretreatment method allows for the extraction of most of the copper and zinc from the sample of flotation tailings [47]. 1.0 kg of the sample was treated with distilled water in a laboratory reactor for 24 h, with constant stirring at 600 rpm, in a volume of 2.5 L. During this process, soluble copper and zinc compounds were removed along with the liquid phase, which can accumulate over time due to electrochemical and biological corrosion of sulfide minerals [48,49]. These compounds can significantly affect the evaluation of the efficiency of metal leaching. After treatment, the sample was separated from the liquid phase using vacuum filtration and mechanical removal of particles with filter paper (pore diameter of 5–8 µm). The filtration precipitate was dried to a constant mass at 80 °C. After that, it was ground in a disk grinder and then sieved through a 1 mm mesh screen. After being dried and ground, a sample of the flotation tailings was used in bioleaching experiments.

### 4.2. Dust Fraction Printed Circuit Boards Sample

**Figure 7:**
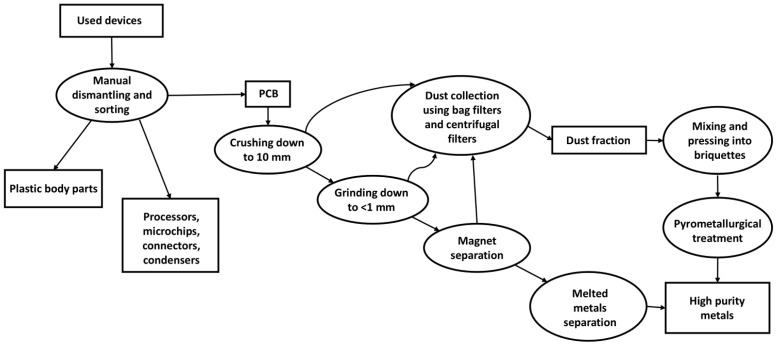
Scheme of recycling of discarded electronic devices by sorting, crushing and subsequent magnetic separation.

In this study, we used the by-product from the mechanical processing of printed circuit boards, including hammer and roller shredders, mills, and grinders, as well as the subsequent homogenization of the resulting powder using a vibrating screen. This process of shredding circuit boards is accompanied by the continuous operation of an air purification system to remove dust generated during the process. The final product of this process, after magnet separation or flotation process [50], is a non-ferrous metal-rich concentrate that is then processed by pyrometallurgical processing (Figure 7). However, the fine dust fraction, which accounts for 2–10% of the total feedstock mass, presents challenges in processing due to its heterogeneity in particle properties, volatility, and low wettability [11,51,52]. This waste material is typically removed manually in traditional circuit board processing technologies. The metal-containing parts are pressed and mixed with various binding materials before being sent for further pyrometallurgical processing [52,53].

The sample of fine dust collected from the air purification system during the mechanical grinding of printed circuit boards, as well as data on the element content in the sample, were provided by RECOMPLETE LLC (Dolgoprudny, Moscow region, Russia) (Table 3).

The uniformity of the electronic waste sample was achieved by separating it using a sieve with a mesh size of 1 mm. By removing large particles from the material, which mainly consist of conductive elements, plastic fragments, and foil, we were able to improve the accuracy and repeatability of our experimental results.

### 4.3. Experimental Setup

The biooxidation experiments were carried out under conditions that were optimized for mesophilic acidophilic microorganisms, including species from the genera *Leptospirillum*, *Acidithiobacillus*, and *Sulfobacillus*. These conditions were selected in order to maximize microbial activity and the chemical kinetics of pyrite oxidation, based on multiple experiments that had been conducted [14,33,53,54,55].

The experiment was carried out in laboratory reactors with a stirrer with a working volume of 1 L, a stirring speed of 500 rpm, constant aeration with an air flow rate of 3 L/min and at a temperature of 35 °C. The nutrient medium used in the experiment was of the following composition (g/L): (NH_4_)_2_SO_4_—0.75, KCl—0.05, MgSO_4_ × 7H_2_O—0.125 and K_2_HPO_4_—0.125. This nutrient medium is widely used in experiments on the leaching of sulfide minerals [33,54,55]. The experiment was conducted in two stages—in a batch and continuous mode. Pulp temperature was maintained using TW-2.03 circulating water baths (Elmi, Riga, Latvia) and U-shaped titanium heat exchangers. RW-20 digital overhead stirrers (IKA, Staufen, Germany) were used for stirring.

#### 4.3.1. Experimental Setup in Batch Mode

The experiment was conducted using two reactor setups in batch mode, with all steps repeated three times. A mineral nutrient medium was acidified with sulfuric acid to a concentration of 2.7 g/L. A total volume of this medium, sufficient to fill both reactors to their operating level, was inoculated with microbial biomass. After thoroughly mixing, the resulting suspension was evenly distributed between the reactors.

**Figure 8:**
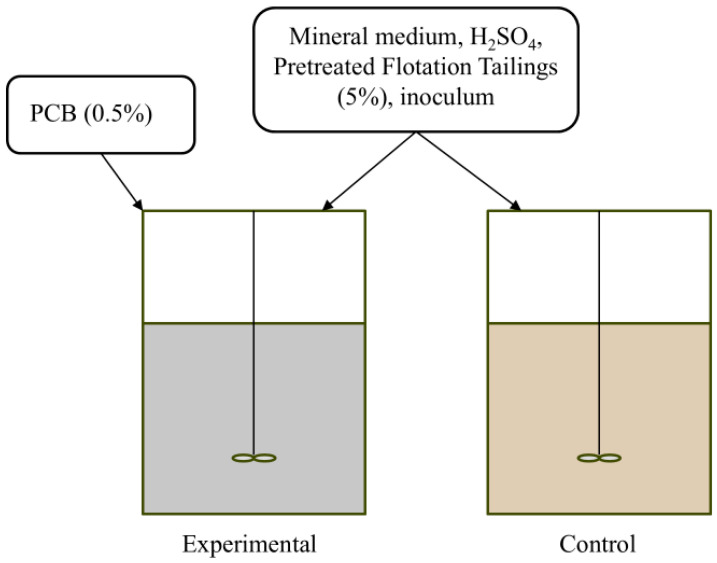
The scheme of the experiment in the batch mode.

To each reactor, a suspension of pretreated flotation tailings was added to achieve a final pulp density of 5% (S:L), which is equivalent to 50 g/L. This constituted the control reactor. For the experimental reactor, a dust fraction from PCBs was also added to the medium at a concentration of 5 g/L alongside the 5% flotation tailings. The configurations of the reactors are illustrated in Figure 8.

The reactors were inoculated to a concentration of 0.5–2.0 × 10^7^ microbial cells/mL. The inoculum was an enrichment culture of acidophilic microorganisms that were maintained at constant aeration and room temperature using pyrite as a source of iron and sulfur. The initial inoculum used in the experiment was not adapted to the specific raw materials of flotation tailings and ground PCB. To prepare the inoculum, it was incubated at room temperature in similar pyrite flotation tailings, with a pulp density of 15%, in a special airlift reactor designed to maintain the conditions for the microbial growth. After that, microbial cells were separated from the pulp using differential centrifugation at 250 g and 10,000 g for 10 min.

Subsequently, the reactors were inoculated with a microbial suspension that was obtained from the corresponding reactors during this experiment.

#### 4.3.2. Experimental Setup in Continuous Mode

**Figure 9:**
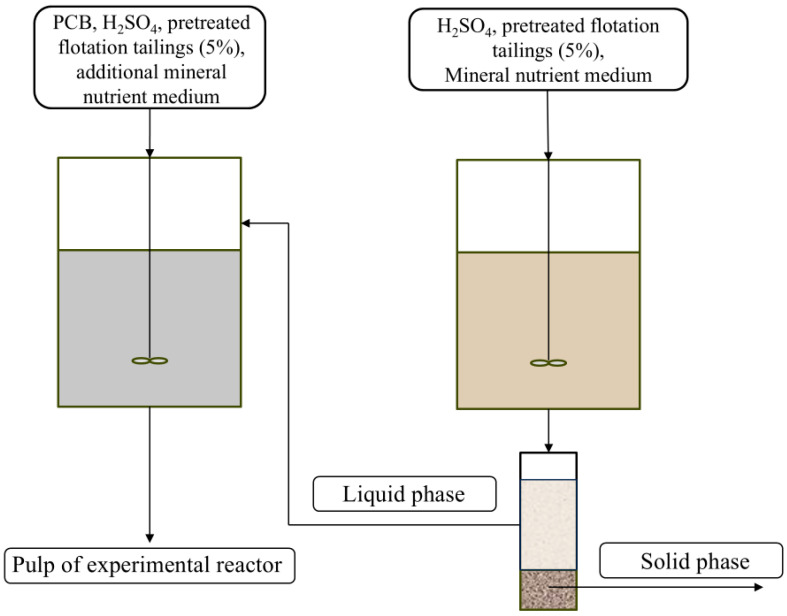
The scheme of the experiment in the continuous mode.

In continuous mode, both reactors operated in a cascade. A flow of mineral nutrient medium, with a portion of pretreated flotation tailings, was fed into the control reactor. The pulp containing oxidized flotation tailings and leaching solution was discharged from the control reactor and separated into two phases using vacuum filtration through filters with pore sizes of 5–8 µm. The separated solid phase was removed, and the liquid phase was fed into the experimental reactor, along with sample of the dust fraction from printed circuit boards and pretreated flotation tailings. The volume of the liquid phase in the experimental reactor was adjusted to the working volume by adding a mineral nutrient medium. Constant flow of leaching solution from the control reactor was created into the experimental reactor, stimulating chemical leaching of PCB dust fraction and continuously supplying microorganisms to the experimental reactor (Figure 9):

In continuous mode, the leaching process was carried out with a retention time of 5 days in each reactor. The same flow rate was used in the experimental reactor using the liquid phase from the control reactor, pretreated PCB dust fraction, flotation tailings and mineral nutrient medium.

During the experiment, the concentration of PCB dust in the pulp was increased in three stages by adding more PCB dust to the feed pulp entering the reactor. The addition rates were 5.0, 12.5, and 37.5 g per liter (g/L) of pulp for the first, second, and third stages, respectively.

The dynamic concentration of PCB dust in the reactor pulp was calculated iteratively using a material balance model in Microsoft Excel (Equation 9). The system volume was maintained constant ($V_{total}$ = 1000 mL). A volume of pulp ($V_{added}$) with a known PCB concentration ($Y_{i}$) displaced by an equal volume of the pulp in the reactor with PCB concentration ($X_{i}$). The new concentration $X_{i+1}$ was calculated from the previous concentration $X_{i}$ and the remaining volume ($V_{remaining}$) as follows (9):

(9) $$X_{i+1}\;=\frac{\left(\left(X_{i}\;V_{remaining}\;+\;Y_{i}\;V_{added}\right)\right)}{V_{total}},$$

The concentration of the PCB dust in the added pulp ($Y_{i}$) was changed stepwise according to the experimental conditions: 5 g/L (days 0–9), 12.5 g/L (days 11–20), and 37.5 g/L (days 22–34). The initial concentration was 5 g/L. The calculated dynamics of $X_{i}$ concentration are shown below (Table 4).

### 4.4. Sampling and Analysis

The concentrations of ferrous and ferric iron were determined by trilonometric titration [56]. The concentrations of Cu^2+^. Zn^2+^. and Ni^2+^ in the samples were measured using atomic absorption spectroscopy using acetylene flame on a Perkin Elmer 3100 atomic absorption spectrometer (Perkin Elmer, Waltham, MA, USA). The samples were pretreated by dilution with 1% HNO_3_. The leaching yield ($Y_{m}$) of non-ferrous metals was calculated according to the concentration of metal in the liquid phase of experimental ($m_{LE}$) and control ($m_{LC}$) reactors, normalized to the total mass of the target metal present in the original PCB dust sample (10):

(10) $$Y_{m}=\frac{m_{LE}-m_{LC}}{X_{i}V_{total}\omega }\times 100\%,$$

where ω is the content of the target metal in PCB sample.

### 4.5. Microbial Population Analysis

Quantitative assessment of microorganisms was carried out by direct counts using an Amplival (Carl Zeiss, Jena, Germany) microscope equipped with a phase-contrast de-vice. The composition of microbial populations that formed during the experiments was determined by the metabarcoding of V3-V4 variable regions of the 16S rRNA gene. fragment using the MiSeq system (Illumina, San Diego, CA, USA). The goal of the microbiological study was to assess the impact of PCB exposure in both batch and continuous modes. A cumulative culture of pyrite-oxidizing microbes served as the inoculum and was sampled prior to the start of the experiment. Biomass samples were also taken at the conclusion of each batch-mode leaching cycle (three times for each reactor in total). During the continuous leaching process, biomass samples were collected before each change in the weight of the ground PCB samples and at the end of the experiment (34 days of the experiment in continuous mode).

The 16S rRNA gene V3-V4 fragment sequences were deposited in the NCBI Sequence Read Archive and are available via the BioProject accession number PRJNA1406024.

### 4.6. Data Processing

During the first stage of the experiment, experiments in control reactor were conducted in batch mode. Data collected from these experiments were analyzed statistically using Microsoft Excel 2015. Standard deviation was calculated based on a standard formula using data from 3 repetitions of the control reactor in a batch mode. Copper and nickel concentrations were also calculated using the same method. In continuous mode, the focus was on prolonged monitoring of the cascade system’s behavior. This allowed us to observe both stable trends and dynamic changes, which were clearly visible in the charts. Since the process was continuous, statistical analysis of individual time points was not possible. Instead, averages and confidence intervals were calculated for each 11–12-day interval, allowing us to visualize trends more clearly over time. The Pearson’s correlation model was also used to analyze the relationship between PCB presence and the relative abundance of different genera of microorganisms.

## 5. Conclusions

This study demonstrates the successful adaptation of an acidophilic microbial community to printed circuit board (PCB) materials during the bioleaching of copper-zinc flotation tailings. During batch mode, community adaptation was evident by a reduction in the lag phase from 24 to 7 days, and consistent metal recoveries of 70 ± 5% for copper and 45 ± 5% for nickel by day 10, across multiple trials. Transitioning to continuous co-leaching mode allowed for stable operation at a PCB pulp density of 1.2%, achieving sustained copper extraction exceeding 80% and nickel recovery of 35–38%. However, increasing the PCB concentration to 35.5 g/L negatively impacted leaching efficiency, indicating a threshold tolerance.

Throughout both operational modes, significant shifts in the microbial community composition were observed, highlighting the dynamic response to PCB presence. It has been shown that the relative abundance of the genus *Ferroplasma* had a negative correlation of −0.77 in the batch mode and −0.72 in the continuous mode, even though it was continuously inoculated with the liquid phase from the control reactor containing cells of these archaea. The relative abundance of the *Sulfobacillus* genus also showed a negative correlation with PCB presence, with correlations of −0.30 and −0.95 for the batch and continuous modes, respectively. These results suggest a possible explanation for the challenges associated with PCB bioleaching, including decreased microorganism activity in one-stage processes. Previous studies have mainly focused on the process itself, rather than shifts in the microbial community caused by PCB. The results obtained will provide new insights into constructing artificial microbial communities with specific acidophiles-mixotrophs adapted to PCB materials that can be used in bioleaching processes. Consequently, future research should focus on artificially assembling microbial consortia resistant to PCB materials in order to optimize the co-leaching process. Promising directions include the selection of supplementary substrates to enhance the survival of key archaea and bacteria, as well as the systematic investigation of cultivation parameters such as temperature, pH, aeration, and carbon supply under varying PCB compositions and the addition of different minerals. In the present work, novel method for effective leaching was proposed, including the use of flotation tailings as a substrate and the cascade of two reactors, with one reactor playing the role of a spent medium generator and inoculator for the second reactor for PCB bioleaching. Based on previous research, the experiment emphasizes the critical importance of microbial diversity and adaptability for process stability.

However, there are still many points that need to be determined. First and foremost, the toxic chemicals of PCB, which play a crucial role in limiting microbial activity, are still undetermined and require further investigation. For instance, the actual behavior of flame retardants in acidic media during the bioleaching process has not been clearly described. Additionally, the desorption process of these components and its dynamics, which can obviously occur in the system, need to be examined. Furthermore, chemical interactions between metals leached from pyrite and other components may occur, and this has not been described in the current work. Another limitation of the study is the use of only one type of PCB sample and flotation tailings, which may not be easily applicable in an industrial setting. Additionally, the PCB pulp density described in the study is relatively low, which could lead to economic limitations in large-scale industrial processes. Future work will focus on expanding the scope of the technological approach by using different types of e-waste samples and increasing the efficiency of the process.

## Figures and Tables

**Table 1:** Results of the experiment in a batch mode. The confidence interval was calculated from 3 runs (*n* = 3) in the control reactor.

Run	Time to Reach the Reference pH Value (1.20), Days	Time to Reach the Reference Eh Value (800 mV), Days	The Time of Complete Oxidation of Ferrous Iron, Days	The Duration of the Lag Phase, Days.
1	29	24	24	24
2	20	15	12	12
3	13	10	10	10
Control (average)	11.3 ± 1.15	10 ± 1.52	9 ± 3.0	7 ± 1.0

**Table 2:** Results of the experiment in continuous mode. The confidence interval was calculated using data obtained from both the control and experimental reactors during the three stages of the experiment, which each lasted 11–12 days.

PCB Concentration, g/L	Reactor	pH	Eh, mV	Fe^3^, g/L	Fe^2+^, g/L	10^7^ Cells/mL	Cu Yield, %	Ni Yield, %
5	Control	1.24 ± 0.10	851 ± 58	10.13 ± 0.3	0 ± 0	201 ± 107	88.95 ± 6.68	48.53 ± 4.31
	Exp.	1.16 ± 0.06	874 ± 11	16.17 ± 3.3	0 ± 0	178 ± 29		
5–12	Control	1.23 ± 0.06	854 ± 52	10.81 ± 0.4	0 ± 0	247 ± 61	83.51 ± 5.91	37.62 ± 6.54
	Exp.	1.16 ± 0.05	863 ± 28	18.23 ± 1.0	0 ± 0	161 ± 50		
12–35.5	Control	1.22 ± 0.08	837 ± 33	8.18 ± 2.3	0 ± 0	132 ± 65	58.39 ± 3.77	27.06 ± 1.62
	Exp.	1.39 ± 0.18	672 ± 26	6.97 ± 3.32	5.24 ± 1.45	17 ± 11		

**Table 3:** The composition of the sample of the dust fraction of the crushing of printed circuit boards analyzed using ICP-AES JY 38 spectrometer (Jobin Yvon, Paris, France).

Content, %													
Si	Fe	Cu	Al	Pb	Zn	Ni	Sn	Cd	Ag	Pd	Rh	Au	Pt
10	8	2.6	2.0	1.7	1.0	0.5	0.5	0.04	0.026	<0.005	<0.002	0.001	<0.001

**Table 4:** Dynamics of PCB dust concentration in the system with stepwise changes.

Stage	Days	Concentration of PCB Dust in the Reactor (X_i_)	Concentration of PCB Dust in the Added Pulp Y_i_
First stage with 5 g/L inlet flow	0	5	5
	3	5	5
	5	5	5
	7	5	5
	9	5	5
Second stage with 12.5 g/L inlet flow	11	5	12.5
	14	8	12.5
	16	9.8	12.5
	18	10.9	12.5
	20	11.5	12.5
Third stage with 37.5 g/L inlet flow	22	11.9	37.5
	25	22.2	37.5
	27	28.3	37.5
	29	32.0	37.5
	31	34.2	37.5
	34	35.5	37.5

## Data Availability

The raw data generated from the 16S rRNA gene profiling are accessible via the BioProject accession number PRJNA1406024.
